# Effect of Capacitive Radiofrequency on the Fibrosis of Patients with Cellulite

**DOI:** 10.1155/2013/715829

**Published:** 2013-10-10

**Authors:** Rodrigo Marcel Valentim da Silva, Priscila Arend Barichello, Melyssa Lima Medeiros, Waléria Cristina Miranda de Mendonça, Jung Siung Camel Dantas, Oscar Ariel Ronzio, Patricia Meyer Froes, Hassan Galadari

**Affiliations:** Potiguar University (UnP), Laureate International Universities, 59054-180 Natal, RN, Brazil

## Abstract

*Background*. Cellulite is a type of lipodystrophy that develops primarily from an alteration in blood circulation or of the lymphatic system that causes structural changes in subcutaneous adipose tissue, collagen, and adjacent proteoglycans. The radiofrequency devices used for cutaneous applications have shown different physiological treatment effects, but there is controversy about the suitable parameters for this type of treatment. *Objectives*. The aim of this study was to evaluate the effects of low-temperature radiofrequency to confirm the thinning of the collagen tissue and interlobular septa and consequent improvement of cellulite. *Methods*. A sample of eight women was used to collect ultrasonographic data with a 12 MHz probe that measured collagen fiber thickness. The Vip Electromedicina (Argentina) device, frequency of 0.55 MHz and active electrode 3.5 cm in diameter (area = 9.61 cm^2^), was applied to a 10 cm^2^ region of the gluteal region for 2 minutes per area of active electrode, during 10 biweekly sessions. *Results*. The Wilcoxon matched paired test was applied using GraphPad InStat 3.01 for Win95-NT software. Pre- and posttreatment mean collagen fiber thickness showed a 24.66% reduction from 1.01 to 0.67 mm. Statistical analysis using the Wilcoxon matched paired test obtained a significant two-tailed *P* value of 0.0391. *Conclusion*. It was concluded that the use of more comfortable temperatures favored a reduction in fibrous septum thickness and consequent cellulite improvement, evidenced by the lower degree of severity and decrease in interlobular septal thickness.

## 1. Introduction

Cellulite is a type of lipodystrophy widely considered as an esthetic disorder in dermal and hypodermal tissue, whose alteration is based on a morphological disorder. It develops primarily from an alteration in blood circulation and of the lymphatic system, causing structural changes in subcutaneous adipose tissue, collagen, and adjacent proteoglycans [1, 2].

In cellulite, fat is stored in fat cells that lie between the skin and muscle tissue. Fat cells are grouped together into large conglomerates separated by fibrous strands (fibrous septae). These fibrous strands run between the muscle and the skin and serve to hold the fat in place (in small compartments). The skin is tethered down by string-like tissues that pull it inward, toward the interior of the body. As fat cells expand with weight gain, the gap between the muscle tissue and skin expands. The fibrous strands cannot stretch and cannot support the skin. The tension of these septae pulls sections of fat in along with them, causing the fat cells in the subcutaneous layer to increase and stick together within the connective tissue fibers, resulting in dimpling (also described as “mattress” or “cottage cheese”) [3].

In the past decade, cellulite management has inspired a new generation of innovative medical devices, such as radiofrequency machines, which promise to correct cellulite signs and symptoms [4]. Radiofrequency energy has been used for more than a century in a variety of medical applications. It is conducted electrically to the tissue, and heat is produced when the inherent impedance of the tissue converts the electrical current to thermal energy. RF devices, which have been used for cutaneous applications, exhibit different physiological effects: neocollagenesis, the lifting effect, decreased localized adiposity, reduced edema and fibroses, and improved cellulite [5–7].

Several experimental in vivo and in vitro studies have produced evidence about the thermal modification of collagen tissue, but there is no consensus about the optimal therapeutic algorithm [5]. At different temperaturas, it is possible to increase or decrease the density of collagen tissue, mainly of fibrous septa found in the cellulite process [8]. When heated, collagen, a very organized crystalline protein structure, transforms into a disorganized gel. Its triple helix shape is destroyed, since its intermolecular bonds are sensitive to low heat. When this disorganized gel is subjected to temperatures above 8 degrees, its structure is transformed into a thicker, rigid tissue, with little fluid and no elasticity [8, 9].

Hence, there are a number of controversies about ideal temperatures for treating cellulite. Some authors propose high temperatures: Alster and Lupton [6] treated cellulite, obtaining immediate collagen contraction due to heat and protein denaturation using high temperatures and del Pino et al. [7] observed a thickening and realignment of interlobular septae using temperatures between 39 and 41°C with radiofrequency. These temperatures are considered high, but there is another authors that uses low temperature, about 37 degrees or 5 to 6 degrees above the temperature of the skin [8, 9].

The problem lies in the fact that most cellulite treatments have proven to be ineffective, since the assessment methods used are mostly subjective or do not provide enough information for the study of subcutaneous tissue. The application of different temperatures to treat cellulite are suggested by several authors [10, 11], as well as differences between their classification. According to Goldman et al. [12], the hard cellulite is typical of young subjects with toned tissues, typically in Latin American people. Normally, the area is rigid and presents adherences between superficial and deep layers and the skin thickness is increased. In these cases, according to some authors [8, 9], the use of low temperatures would be more interesting to refine fibrous septae. On the other hand, soft cellulite is common in older people and sedentary, with characteristics of weak and white skin. In this case, radiofrequency high temperature increases the collagen thickness.

High-resolution ultrasound allows the observation of subcutaneous tissue, the fat located between skin and muscle, and the anatomic views of the layer between the subcutaneous tissue and the adipose layer, as well as the integrity of the fibrous bands (fibrous septa) that divide them [7].

Because of the aforementioned problem, the aim of this study was to evaluate the effects of low-temperature radiofrequency (5 to 6 degrees above skin temperature) in hard cellulite, to confirm the thinning of the collagen tissue and interlobular septa and consequent improvement of cellulite.

## 2. Material and Methods

The study was approved by the Human Research Ethics Committee of Universidade Potiguar. The sample was composed of eight women volunteers selected according to the following inclusion criteria: age between 25 and 40 years and complaint of grade 2 and 3 cellulite (according to Curri's classification) [12], located in the gluteal region, and willingness to submit to the treatment. Pregnant and diabetic women undergoing drug or hormone treatment were excluded. The participants were not submitted to any food restriction and were asked to maintain their usual daily activities. After being informed about the purpose of the study and the procedures that would be followed, the women gave their written informed consent.

The following data collection instruments were used: Fibro Edema Geloid Assessment Protocol (PAFEG), proposed by Meyer et al. [13] A Sony digital camera, 7.2 megapixels; GE Vivid 3 ultrasound machine, with multifrequency (6.0–16 MHz) probe; radiofrequency device and infrared digital thermometer were also used.

An evaluation was conducted based on PAFEG, which is composed of three items: identification, anamnesis, physical examination containing inspection and palpation, topographic location, severity classification, tactile sensitivity test, and complementary examinations associated with general patient information. An ultrasonographic examination was performed by a medical specialist using a 12 MHz probe to measure collagen fibers before and after radiofrequency treatment. This examination is a simple, nonintrusive, and reliable method that enables measuring the thickness of interlobular septae present in more advanced cellulite. Three different septae located at the center of the demarcated area were measured pre- and posttreatment with a pendulum probe to avoid influencing the measures, and the resulting values of these parameters were used for statistical analysis. The demarcated area remained during the entire treatment and adipose layer thickness was also measured both brfore- and after treatment. GraphPad InStat 3.01 for Win95-NT software and the Wilcoxon matched paired test were used for the analyses.

Radiofrequency was applied to the gluteal region with the volunteers in the ventral decubitus position and the lower extremities extended and relaxed. After delimitating an area of 10 cm^2^ and performing asepsis (70% alcohol) of the area to be treated, we applied the Tecartherap-Vip, Vip Electromedicina (Argentina) radiofrequency capacitive device, with frequency of 0.55 MHz, active electrode 3.5 cm in diameter (area = 9.61 cm^2^), and passive metal electrode with an area of 240 cm^2^, placed in the lower abdominal region with Carbopol gel for coupling. The application zone was divided by the measure of the active electrode, obtaining the measure of 8 electrodes. After the patient's skin temperature was measured, the application was initiated, continuing until the temperature was 5 degrees above the initial value. Once this level was reached, linear movements (back and forth) were performed for 2 minutes on the area, relative to the size of two electrodes, during 10 biweekly sessions.

Statistical analysis of collagen fiber thickness before and after treatment was based on the PAFEG data and ultrasonographic examination results obtained.

## 3. Results

**Figure 1:**
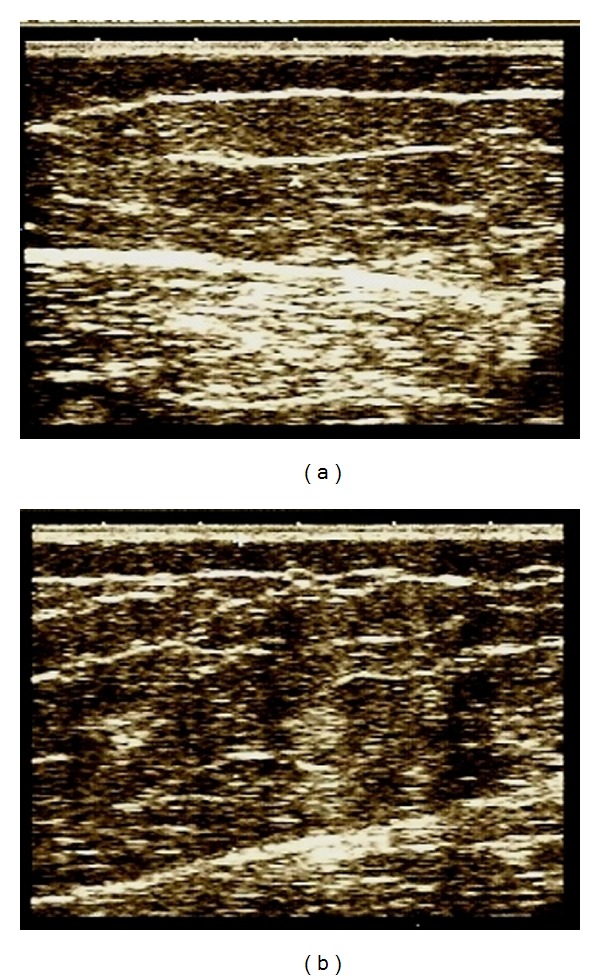
Ultrasonography before and after 10 sessions.

**Figure 2:**
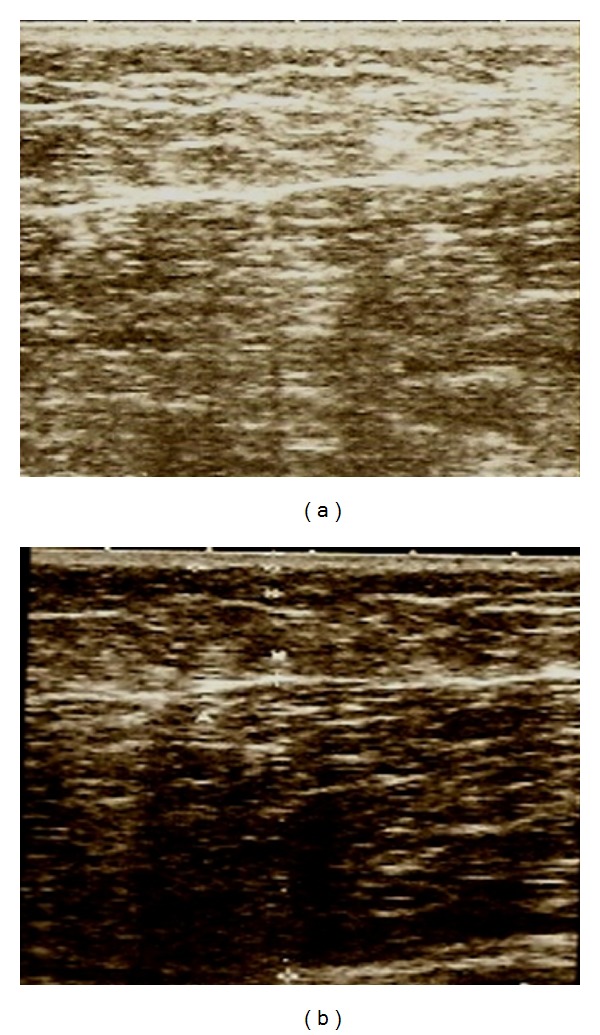
Ultrasonography before and after 10 sessions.

**Figure 3:**
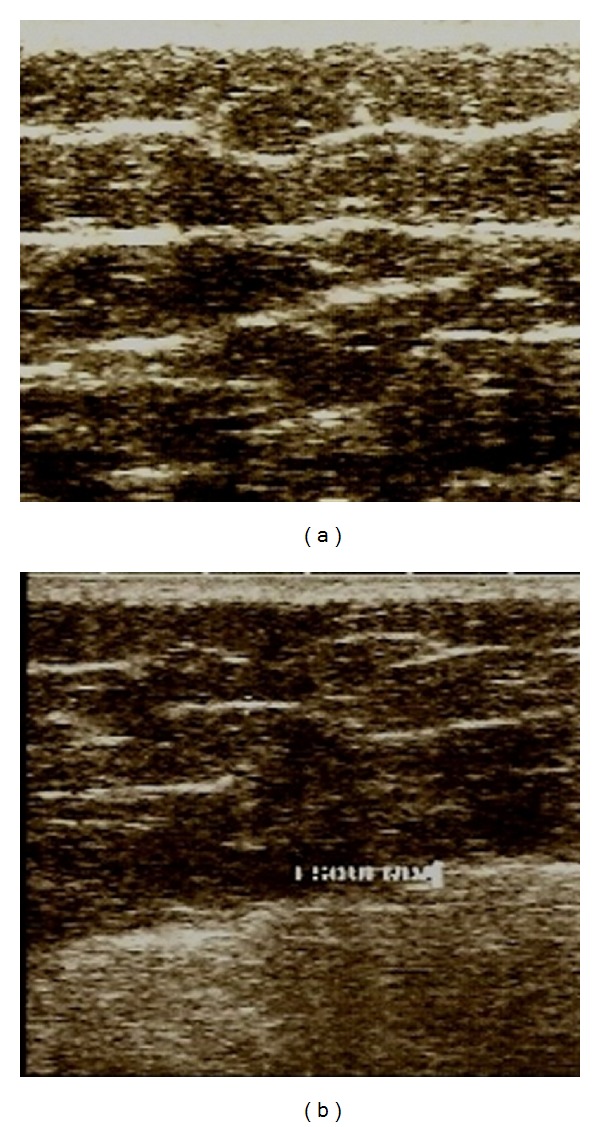
Ultrasonography before and after 10 sessions.

**Figure 4:**
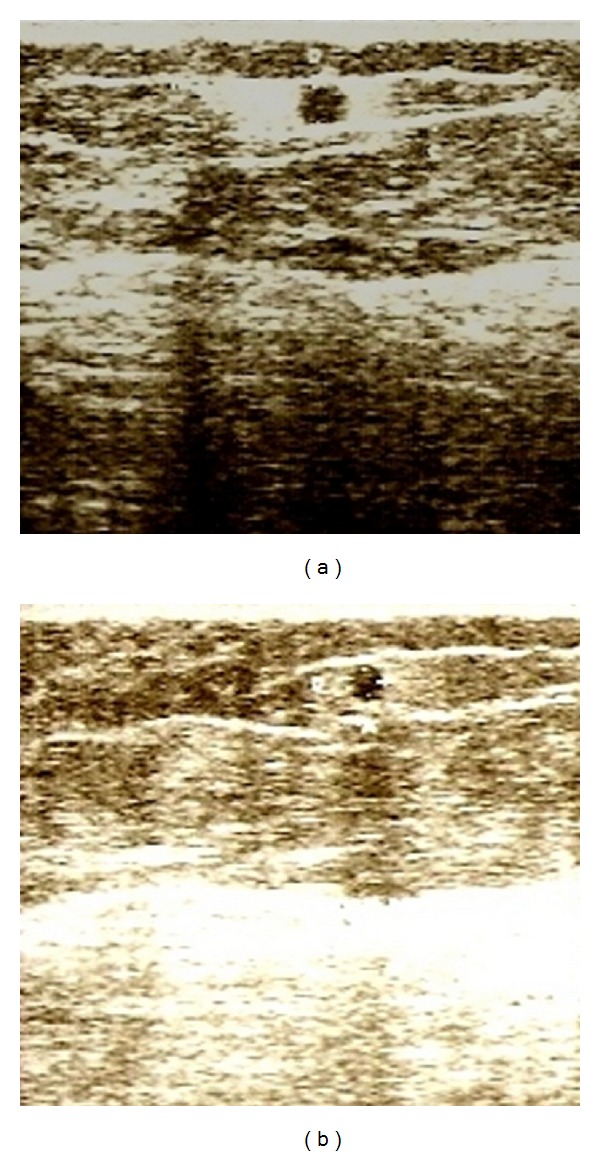
Ultrasonography before and after 10 sessions. These figures shows the presence of an adipose tissue graft performed 4 years before following lipoaspiration. The tissue is surrounded by interstitial fibrosis in (a), but after treatment (b) there is no visible fibrosis in this area.

 Figures 1, 2, 3, and 4 correspond to the organization and deposition of fibrous septae in cellulite areas before and after radiofrequency treatment.

 The mean thickness values obtained for 3 fibrous septae are shown in Table 1. 

 The mean pre- and posttreatment collagen fibrous thickness was 1.01 mm and 0.67 mm, respectively, a reduction of 24.66%. Statistical analysis using the nonparametric test for paired data (Wilcoxon matched paired test) showed a significant two-tailed *P* value of 0.0391.

Adipose layer thickness results are shown in Table 2. 

The mean pre- and posttreatment adipose tissue layer thickness was 29.76 mm and 30.31 mm, respectively, an increase of 0.55 mm (5.51%). Statistical analysis using the nonparametric test for paired data (Wilcoxon matched paired test) obtained a nonsignificant two-tailed *P*-value of 0.6406. This finding is correlated with the increased weight of most of the patients (mean of 0.51 Kg) (Table 3).

 The cellulite showed altered conjunctive and adipose tissue disposition, with adipose and cell hyperplasia and hypertrophy, as well as polymerization of the fundamental amorphous substance, resulting in proliferation of intra-adipocyte and interlobular collagen fibers. These alterations provoke reduced circulation in tissues, reduced drainage and fibroblast incarceration, and enrichment and rupture of elastic fibers [14].

 Figures 1 to 3 show the ultrasonographic results of collagen fiber thickness after radiofrequency treatment at comfortable temperatures (5 to 6 degrees above skin temperature), demonstrating a decrease in interstitial fibrosis. Two temperature observation parameters can be used to apply radiofrequency: the first is based on infrared thermometer values and the second based on the subjective scale of heat applied to each patient. It is suggested that G3, a moderate and pleasant heat level, be reached to achieve an increase in the distensibility of collagen tissue [8].

Figure 4 shows the presence of an adipose tissue graft performed 4 years before following lipoaspiration. The tissue is surrounded by interstitial fibrosis in Figure 4(a), but after treatment (Figure 4(b)) there is no visible fibrosis in this area. The reorganization of fibrous tissue, as well as the reduction in fiber thickness observed after ultrasonographic examination, may be associated with the effects of radiofrequency. According to Verrico et al. [15], radiofrequency thermotherapy favors the absorption of type I collagen by activating protein metabolism activators. This corroborates the results and biological effects obtained for fibrous septum thickness as well as the improvement in cellulite. 

## 4. Discussion

The radiofrequency effects on the conjunctive tissue evaluated by ultrasonography have been documented in a number of studies [6, 7]. In research using high temperatures, the changes observed reflect the increased echodensity of conjunctive tissue structures, evidenced by an increase in the amount of fibers and compactness of existing fibers. This allows us to assume that high-temperature RF worsens the clinical picture of cellulite.

According to the literature, radiofrequency also favors a decrease in lipolysis and thickness and fat accumulation in adipocytes, consequently reducing venous and lymphatic fluid retention caused by hypodermal tissue compressing vessels and nerve endings [16]. Table 3 shows that the temperature conditions of this study did not satisfactorily alter adipose tissue measures, evidenced by oscillating increases and decreases in these values. According to PAFEG assessment, the anthropometric data of the patients changed considerably during the study, influencing the reduction of adipose tissue. It should be pointed out that no changes in patient diet were indicated.

It was demonstrated that ultrasonography can be used as a diagnostic method to evaluate the characteristics of subcutaneous tissue and to observe the effects of radiofrequency in cellulite treatment. This methodology could be applied to evaluate other treatments related to cellulitis and localized fat. A difficulty of this study was a small sample because of the cost of the ultrasonography exam, so we suggest the repetition of this research with a greater number of patients.

The use of more comfortable temperatures favored both a reduction in fibrous septum thickness and an improvement in the clinical appearance of cellulite, demonstrated by the reduced degree of severity and decreased interlobular septal thickening. Radiofrequency has been little studied in the area of esthetic medicine; therefore, more studies are needed to ascertain its real effects, since temperature variations significantly affect collagen tissue.

## Figures and Tables

**Table 1:** Average thickness of collagen fibers.

Patient	Grade of cellulitis	Average thickness of collagen fibers (measures/3)			
		Before treatment (mm)	After treatment (mm)	Difference (mm)	Difference Porcentual (%)
1	Grau 2	1.25	0.60	−0.65	−52.00
2	Grau 2	0.73	0.56	−0.17	−23.29
3	Grau 3	1.13	0.70	−0.43	−38.05
4	Grau 2	0.56	0.55	−0.01	−1.79
5	Grau 3	1.92	0.75	−1.17	−60.94
6	Grau 2	0.70	0.60	−0.10	−14.29
7	Grau 3	0.63	0.80	0.17	26.98
8	Grau 3	1.15	0.76	−0.39	−33.91
	Media	**1.01**	**0.67**	**−0.34**	**−24.66**

**Table 2:** Average of adipose tissue layer.

Patient	Grade of cellulitis	Average of adipose tissue layer			
		Before treatment (mm)	After treatment (mm)	Difference (mm)	Difference Porcentual (%)
1	Grau 2	17.0	16.5	−0.5	−2.94
2	Grau 2	22.0	35.5	13.0	59.1
3	Grau 3	33.5	50.7	17.2	51.3
4	Grau 2	28.0	29.0	1.0	3.5
5	Grau 3	35.15	36.8	1.65	4.7
6	Grau 2	21.85	22.8	0.95	4.3
7	Grau 3	33.95	16.4	−17.5	−51.5
8	Grau 3	46.6	35.2	−11.4	−24.4
	Media	**29.7**	**30.3**	**0.5**	**5.5**

**Table 3:** Mean body weight alterations after radiofrequency treatment.

Patient	Alterations in weight (Kg)
1	−1.0
2	1.7
3	2.8
4	0.5
5	0.4
6	1.8
7	−2.1
8	0.0
Media	**0.51**
